# Phenyl-2-aminoethyl selenide ameliorates hippocampal long-term potentiation and cognitive deficits following doxorubicin treatment

**DOI:** 10.1371/journal.pone.0294280

**Published:** 2023-11-10

**Authors:** Ahmad H. Alhowail, Matthew Eggert, Jenna Bloemer, Priyanka D. Pinky, Lauren Woodie, Subhrajit Bhattacharya, Dwipayan Bhattacharya, Manal A. Buabeid, Bruce Smith, Muralikrishnan Dhanasekaran, Gary Piazza, Miranda N. Reed, Martha Escobar, Robert D. Arnold, Vishnu Suppiramaniam

**Affiliations:** 1 Department of Drug Discovery and Development, Auburn University, Auburn, AL, United States of America; 2 Department of Pharmacology and Toxicology, College of Pharmacy, Qassim University, Buraydah, Kingdom of Saudi Arabia; 3 Department of Nutrition, Dietetics and Hospitality Management, College of Human Sciences, Auburn University, Auburn, AL, United States of America; 4 College of Pharmacy and Health Sciences, Ajman University, Ajman, UAE; 5 Department of Anatomy, Physiology and Pharmacology, College of Veterinary Medicine, Auburn University, Auburn, AL, United States of America; 6 Center for Neuroscience Initiative, Auburn University, Auburn, AL, United States of America; 7 Department of Psychology, Oakland University, Rochester, MI, United States of America; 8 Department of Molecular and Cellular Biology, College of Science and Mathematics, Kennesaw State University, Kennesaw, Georgia; University of Nebraska Medical Center College of Medicine, UNITED STATES

## Abstract

Chemotherapy-induced memory loss (“chemobrain”) can occur following treatment with the widely used chemotherapeutic agent doxorubicin (DOX). However, the mechanisms through which DOX induces cognitive dysfunction are not clear, and there are no commercially available therapies for its treatment or prevention. Therefore, the aim of this study was to determine the therapeutic potential of phenyl-2-aminoethyl selenide (PAESe), an antioxidant drug previously demonstrated to reduce cardiotoxicity associated with DOX treatment, against DOX-induced chemobrain. Four groups of male athymic NCr nude (nu/nu) mice received five weekly tail-vein injections of saline (Control group), 5 mg/kg of DOX (DOX group), 10 mg/kg PAESe (PAESe group), or 5 mg/kg DOX and 10 mg/kg PAESe (DOX+PAESe group). Spatial memory was evaluated using Y-maze and novel object location tasks, while synaptic plasticity was assessed through the measurement of field excitatory postsynaptic potentials from the Schaffer collateral circuit. Western blot analyses were performed to assess hippocampal protein and phosphorylation levels. In this model, DOX impaired synaptic plasticity and memory, and increased phosphorylation of protein kinase B (Akt) and extracellular-regulated kinase (ERK). Co-administration of PAESe reduced Akt and ERK phosphorylation and ameliorated the synaptic and memory deficits associated with DOX treatment.

## 1. Introduction

Chemotherapy is an effective treatment for many cancers; however, it can have significant negative side effects. Cancer patients typically report fatigue, pain, loss of appetite, and nausea, following chemotherapy treatment [1]. Furthermore, cognitive deficits have also been reported in up to 75% of patients receiving chemotherapy [2]. These cognitive impairments have become so ubiquitous in chemotherapy treatment that they have earned the name “chemobrain.” Several studies on human patients and animal models have provided evidence that chemotherapy treatment can lead to deficits in key aspects of short-term cognition, such as spatial and fear memory both during and shortly after chemotherapy [3]. However, other studies indicate that there are no significant cognitive effects following chemotherapy [4]. Thus, it is imperative to investigate the effects of chemotherapy on cognition to fully understand this crucial topic and determine the conditions under which cognitive impairment is a consequence of chemotherapy treatment.

Doxorubicin (DOX) is an anthracycline used widely to treat different cancers, including breast, prostate, and lung cancers, as well as osteosarcoma [5]. While DOX is effective against cancer growth, its use is limited by several well-known side effects that include cardiotoxicity, nephrotoxicity, and hepatotoxicity [6–10]. Recently, DOX has also been associated with chemobrain [11]. Though the exact mechanisms by which DOX induces cognitive deficits are still unclear, a few studies have attempted to investigate the molecular mechanisms associated with chemotherapy-induced memory function [10,12–15]. Our recent study suggests one mechanism by which chemotherapy induces memory deficits is *via* alterations in hippocampal synaptic plasticity, as well as synaptic protein signaling [16]. In this current study, we sought to determine whether these DOX-mediated alterations could be prevented by co-administration of phenyl-2-aminoethyl selenide (PAESe).

PAESe is an antioxidant drug previously demonstrated to reduce cardiotoxicity associated with DOX treatment [17]. Though the molecular mechanism by which PAESe exerts antioxidant activity is not clear, it does contain selenium at its core [18]. Selenium is an important element that binds to a number of antioxidant enzymes, including glutathione peroxidase, to enhance their antioxidant activity [19]. PAESe was initially developed to treat hypertension and cardiovascular disease [18], and it was recently found to have anti-tumor and cardioprotective effects [20], as well as the ability to reduce oxidative stress [19,21].

In the present study, the therapeutic potential of PAESe in ameliorating the cognitive and synaptic deficits caused by DOX treatment was investigated in mice. In addition, Western blot analysis was used to elucidate the potential mechanisms associated with DOX-induced cognitive decline. A previous study in rats using the novel place recognition task also showed that DOX impairs spatial memory [22]. Thus, in this study, cognitive function was assessed using both Y maze and Novel Object Location tests. The Y maze is a well-established procedure that can be used to assess working and spatial reference memory in mice [23] and assess hippocampal function deficits [24]. The Novel Object Location test can be used to assess spatial working memory in rodents [25], and it is strongly associated with CA1 activity in the hippocampus [26].

## 2. Materials and methods

### 2.1. Animal treatment

A total of 32 six-week-old male athymic NCr nude (nu/nu) mice (weight range: 25-30g; Taconic Biosciences, Tarrytown, NY) were housed in pathogen-free conditions with a 12 h light/dark cycle and free access to food and water. Male mice were used to be consistent with our prior studies [15,16]. All procedures were carried out in accordance with protocols approved by the Auburn University Institutional Animal Care and Use Committee (IACUC). All euthanasia procedures were in accordance with the guidelines of the American Veterinary Medicine Association [27]. Mice were assigned randomly to one of four groups (*n* = 8 per group) and treated with five weekly intravenous (i.v.) tail-vein injections over a 4-week study period, as follows: (1) control group, 0.9% *w*/*v* sterile saline, (2) DOX group, 5 mg/kg DOX, (3) PAESe group, 10 mg/kg PAESe, and (4) DOX+PAESe group, 5 mg/kg DOX and 10 mg/kg PAESe. Both DOX and PAESe were prepared in 0.9% sterile saline and sterile filtered (0.2 micron filter). DOX was purchased from Sigma-Aldrich (St. Louis, MO), and PAESe was synthesized, as previously described [28], and provided by Dr. Sheldon May (Georgia Tech).

### 2.2. Assessment of spatial memory

The Y-maze was used to assess working memory and spatial memory functions in mice, as described previously [29]. The apparatus for the Y-maze test was composed of three plastic arms (7.5 cm wide x 38 cm long x 15 cm high) at 120° to each other. The maze was placed in the center of a room, ensuring that all arms were equally illuminated, and with a constant white noise as a background that helped mask environmental sounds. Landmarks (geometric figures) were placed 5 cm above the available arms. Animals were 10 weeks of age at the initiation of the training sessions. The training sessions were 15 min in duration, during which time the animals could explore only two arms: the arm where they were placed (the entry arm) and one of the other two arms (the known arm) placed at the left or right of the entry arm. The third arm (the novel arm) was occluded by an opaque divider. The identity of the entry, known, and novel arm was counterbalanced within groups. Animals were then returned to their home cages for 3 h. After this time had elapsed, the animals were placed back in the maze, but this time all arms were open. They were allowed to explore the maze for 10 min and then returned to their home cages. The maze was thoroughly cleaned after each training and testing session with a 35% (*v/v*) alcohol solution to eliminate odor cues. The test session was recorded by video camera and later scored for the number of entries and cumulative time spent in each arm (dwell time). Percent dwell time in the novel arm (*DwellN*) was calculated as a proportion of all time spent in either the novel or the familiar arms (*DwellF*, average of dwell time in the entry and known arm), using the formula, DwellN/(DwellN+DwellF)*100. A dwell discrimination index was also calculated using the formula, *(DwellN-DwellF)/(DwellN+DwellF*), with positive indexes reflecting higher *DwellN* than *DwellF*, negative indexes reflecting the opposite pattern, and zero indexes reflecting no discrimination. Percent number of entries in the novel arm, as well as an entry discrimination index were calculated in a similar manner.

### 2.3. Assessment of working memory

The novel object location (NOL) test is a working memory task that mainly relies on cortical and hippocampal functions. To perform this test, a transparent open box (45 cm × 45 cm × 45 cm) and a video recording system were used. Each side of this box had a different landmark cue adhered to the wall at a height of 30 cm. The box was located in a dimly illuminated room with a constant white noise background. During the first day, mice were given one habituation session (10 min) to the box without any objects present. The next day, two identical objects (two cylinders, 5.5 cm high, 5.5 cm in diameter) were placed adjacent to each other. Mice were placed in the middle of the box and allowed to explore the two objects for 10 min. The mice were returned to their home cages for 1 h and then placed back into the box; however, during this session, one of the objects was moved 90° from the original position (the original position, the object that was moved, and the movement direction were counterbalanced within groups). Mice were allowed to explore the objects for 10 min. Animal activity directed at the objects (contact, manipulation, sniffing, and movement in the direction of the object) occurring in 2.5 cm around the object (i.e., a total area with a radius of 10 cm including the object in the center) was scored as exploration; this resulted in two scores: novel location exploration (*NLE*) and old location exploration (*OLE*). Percent time exploring the novel location was calculated as a proportion using the formula NLE/(NLE+OLE)*100. A discrimination index was also calculated using the formula, *(NLE-OLE)/(NLE+OLE*), with positive indexes reflecting higher *NLE* than *OLE*, negative indexes reflecting the opposite pattern, and zero indexes reflecting no discrimination [30]. One subject in the DOX group did not approach the right side of the box, and all of this subject’s exploration time was limited to the new object location side, and its percent exploration score was a significant outlier for its group (*Z* = 2.02, critical value of *Z* = 1.89). Thus, scores for this subject were excluded from the analyses.

### 2.4. Preparation of the acute hippocampal slices

Animals were euthanized by CO_2_ inhalation and then decapitated for brain collection. A Leica VT1200 S Automated Vibratome (Leica Biosystems Inc., Buffalo Grove, IL, United States) was filled with an oxygenated cutting solution (in mM: NaCl 85, KCl 2.5, MgSO_4_ 4.0, CaCl_2_ 0.5, NaH_2_PO_4_ 1.25, NaHCO_3_ 25, glucose 25, sucrose 75, kynurenic acid 2.0 and ascorbate 0.5) and 350 μm hippocampal slices were obtained. The slices were allowed to rest in a holding chamber, submerged in oxygenated artificial cerebral spinal fluid (aCSF) containing (in mM) 124 NaCl, 3 KCl, 1.5 MgSO47H20, 1.2 NaH2PO4, 2.4 CaCl2, 25 NaHCO3, and 10 D-Glucose, for 2 h at 30°C prior to extracellular field recordings [31].

### 2.5. Extracellular field recordings

Slices were transferred into a submerged recording chamber. The chamber was continuously perfused with oxygenated ACSF (33°C) with a flow rate of 2 ml/min. A platinum bipolar electrode was placed on the CA3 region of the hippocampus, and a glass microelectrode (1.5 mm outer diameter; World Precision Instruments, Sarasota, Florida) filled with ACSF was placed on the stratum radiatum in the CA1 region of the hippocampus to record field excitatory postsynaptic potentials (fEPSPs) from the Schaffer collateral circuit. The CA3 was stimulated with a Model 4D Digital Stimulus Isolation Amplifier (SIU) instrument at 0.05 Hz for 10 min to monitor basal synaptic transmission. LTP was induced using five theta burst stimulations (TBS) with an inter-TBS interval of 20 s. Each TBS consisted of 10 bursts delivered at 5 Hz, with each burst containing four 0.2-ms pulses at 100 Hz. Field potentials were recorded using LTP software with Axoclamp 2B (Axon Instruments, Foster City, CA) and analyzed using WinLTP software [32].

### 2.6. Western blot analysis

Hippocampi from the four treatment groups were dissected and homogenized with Thermo Scientific N-PER Neuronal Protein Extraction Reagent including proteases inhibitors. The homogenates were centrifuged for 15 min at 12,000 g at 4°C and the supernatant was collected. The protein concentrations were determined using the bicinchoninic acid (BCA) protein assay (Pierce, Rockford, IL). Each sample was mixed with 4× Laemmli buffer and loaded into a 10% sodium dodecyl sulphate–polyacrylamide gel electrophoresis (SDS-PAGE) gel. The proteins were transferred to polyvinylidene fluoride (PVDF) membranes (Immobilon-P Millipore, Germany) and blocked by 5% non-fat dry milk for 2 h in Tris-buffered saline containing 0.1% Tween 20 (TBST). Membranes were washed with TBST and incubated with anti-phospho-protein-kinase-B (anti-phospho-Akt), anti-Akt, anti-extracellular-signal-regulated-kinase-1/2 (anti-ERK1/2), anti-phospho-ERK1/2 overnight at 4°C. All primary antibodies were purchased from Cell Signaling Technology, Danvers, Massachusetts (Akt Antibody, Catalog number-9272; Phospho-Akt (Ser473) (193H12) rabbit mAb, catalog number-4058; p44/42 MAPK (Erk1/2) (137F5) rabbit mAb, catalog number-4695; Phospho-p44/42 MAPK (Erk1/2) (Thr202/Tyr204) Antibody, catalog number-9101), and used at a 1:1000 dilution. Membranes were then probed with a secondary anti-rabbit antibody (1:5000) conjugated with fluorophore DyLight 550 at room temperature (25°C) for 1 h. Then, the membranes were scanned and visualized with a FluorChem Q System imager (Proteinsimple, San Jose, California, USA). Following probing for either Phospho-AKT or Phospho-p44/42, the blots were treated with a stripping buffer (Thermo Scientific™ Restore™ PLUS Western Blot Stripping Buffer) and reprobed for total AKT or p44/42, respectively. GAPDH was used as a loading control on a separate gel. Density of immunoreactivity for each band was measured using AlphaView software (ProteinSimple).

### 2.7. Statistics

Data were analyzed using a 2 (DOX treatment) x 2 (PAESe treatment) analysis of variance (ANOVA), repeated measures ANOVAs (RMANOVA), or Kruskal-Wallis tests. Significant omnibus tests are reported in the results section and were followed by planned comparisons. These planned comparisons included contrasts of all groups (Control, PAESe, and DOX+PAESe) to the DOX group, as well as a comparison of the Control vs. PAESe group to determine whether PAESe alone had consequences. These planned comparisons are shown visually in the figures with a * representing a significant difference relative to the DOX group and $ representing a difference between Controls and PAESe groups. A *p*-value < 0.05 was considered significant. All data are presented as means ± SEM.

## 3. Results

### 3.1. PAESe ameliorates behavioral deficits caused by doxorubicin

**Fig 1:**
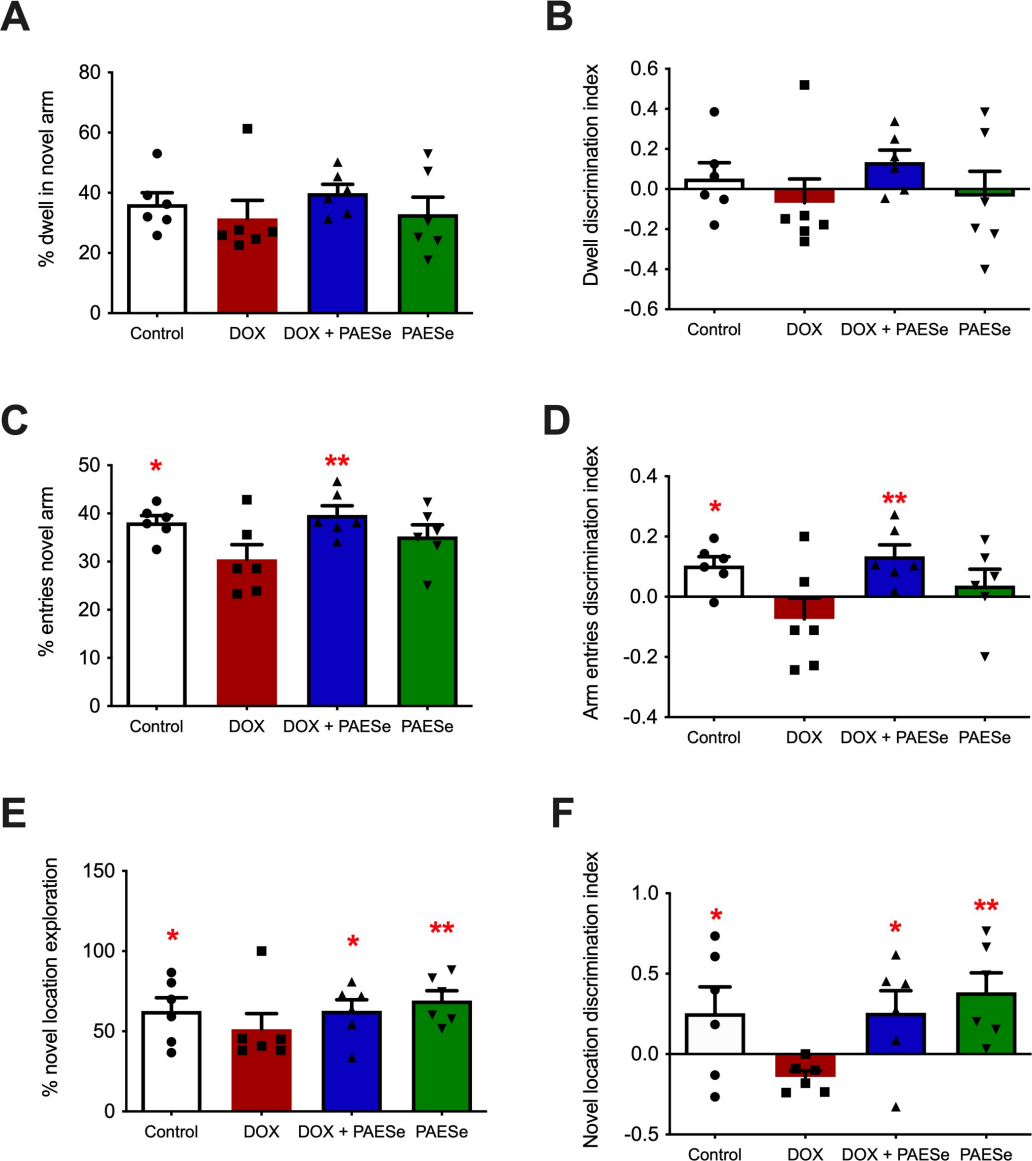
**PAESe ameliorates behavioral deficits caused by doxorubicin treatment.** Spatial memory via a Y-maze test (**A-D**) or working memory via a NOL test (**E-F**) was assessed following treatment with vehicle control (saline), DOX only, PAESe only, or the co-administration of DOX and PAESe. **A:** Percent dwell in the novel arm; **B:** Dwell discrimination index; **C:** Percent entries into the novel arm; **D:** Arm entries discrimination index. Percent scores were calculated as a proportion of total time spent in (dwell) or number of entries into (entries) the novel vs. familiar arms of the maze. Positive discrimination indices reflect more time spent (dwell) or more entries into (entries) the novel than the familiar arms of the maze; negative discrimination indices reflect the opposite pattern, and of zero discrimination indices reflect no discrimination. **E:** Percent time spent exploring the novel object location; **F**: Novel location discrimination index. Percent exploration scores were calculated as a proportion of total time spent exploring the objects in the old and novel locations. Positive discrimination indices reflect more time spent (dwell) or more entries into (entries) the novel than the familiar arms of the maze; negative discrimination indices reflect the opposite pattern, and of zero discrimination indices reflect no discrimination. Bars represent mean ± SEM; n = 5-6/group, * = p < .05 and ** = p < .01 represent a significant difference when compared to the DOX group.

Neither percent dwell time in the novel arm (Fig 1A; *F*s(1,20) < 1.5, *p*s > .2) nor dwell discrimination index (Fig 1B; *F*s(1,20) < 2.2, *p*s > .16) differed among the groups, suggesting neither DOX nor PAESe altered exploratory behavior. However, DOX reduced the percent entries into the novel arm relative to controls, an effect ameliorated by co-administration of PAESe (Fig 1C; DOX x PAESe interaction, *F*(1, 20) = 7.14, *p* < .05). Likewise, the DOX-mediated reduction in the entry discrimination index was restored following co-administration of PAESe (Fig 1D; DOX x PAESe interaction, *F*(1, 20) = 7.40, *p* < .05). Administration of PAESe alone did result in a slight reduction in the percent entries and entry discrimination index, but was not statistically significant from the control group or DOX alone (Fig 1C and 1D).

Similar to the y-maze, DOX-treated animals exhibited deficits in percent novel location exploration in the NOL test relative to both controls and PAESe-treated animals, and PAESe co-administration rescued the DOX-mediated reduction (Fig 1E; main effects of DOX and PAESe treatments, *F*s(1,19) > 4.50, *p*s < .05). Consistent with the observations of percent exploration, the discrimination index was also reduced by DOX treatment relative to controls and PAESe-treated animals, an effect attenuated by co-administration of PAESe (Fig 1F, main effects of DOX and PAESe treatments, *F*s(1, 19) > 4.50, *p*s < .05). Together, these findings suggest DOX treatment impairs the response to novelty (number of entries) in the y-maze and impairs working memory in the NOL task, and that PAESe co-administration attenuates these effects.

### 3.2. PAESe rescues impaired synaptic plasticity in the hippocampus caused by treatment with doxorubicin

**Fig 2:**
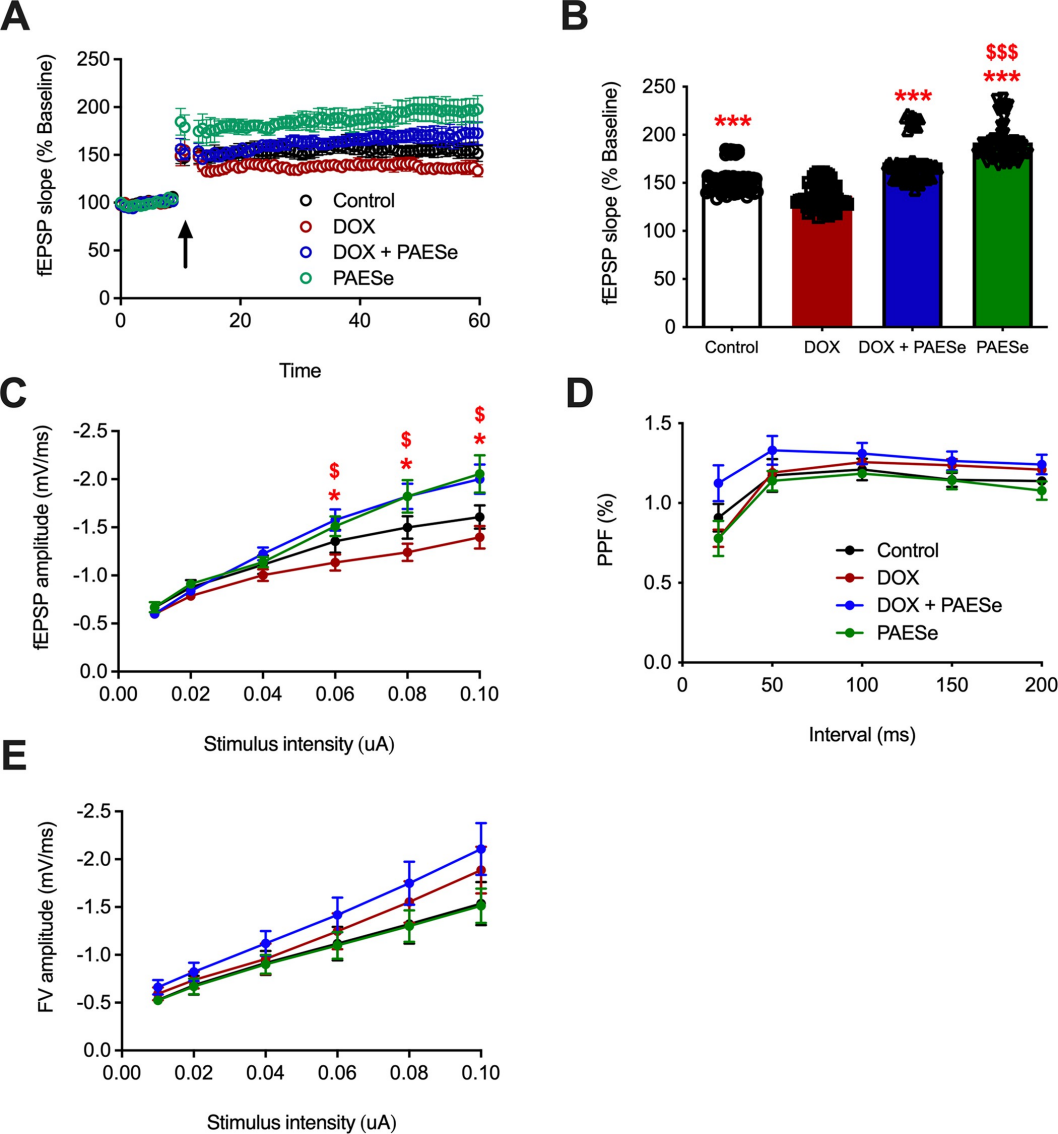
Doxorubicin-treated mice display a reduction in long-term potentiation (LTP) that is rescued with co-administration of PAESe. **A:** LTP graph represents the fEPSP slope before and after induction by HFS, as indicated by the arrow. **B:** LTP bar graph showing fEPSPs recorded for 50–60 min post TBS induction and normalized to baseline levels. **C:** Input output curve depicting fEPSP slope across a range of stimulus intensities. D: PPF measured at different interpulse intervals. **E:** average FV amplitude for each stimulus intensity. Bars represent mean ± SEM; *n* = 5–6 of animals/group, * *p <* .05, ***p < .001 represent a significant difference when compared to the DOX group. $ *p* < .05, $ $ $ *p* < .001 represent a significant difference between the Control vs. PAESe groups.

To determine whether PAESe can ameliorate DOX-induced impairments in synaptic plasticity, we measured LTP in the Schaeffer collateral pathway using hippocampal slices (Fig 2A and 2B; H(3) = 151, *p* < .0001). As we have previously reported [16], DOX-treated mice displayed LTP deficits after high-frequency stimulation (HFS) (mean for control 154.42% vs. 136.60% for DOX-treated animals), and PAESe treatment rescued these deficits in LTP (136.60% for DOX-treated animals vs. 171.35% for DOX+PAESe-treated animals). PAESe alone also increased LTP relative to controls (192.04% for PAESe-treated animals vs. 154.42% for controls).

We next examined changes in basal synaptic transmission by comparing the slope of fEPSP across a range of stimuli and observed differences among the groups with increasing intensities (Fig 2C; RMANOVA intensity x PAESe interaction, *F*(5,85) = 8.7, *p* < .0001). Notably, basal synaptic transmission between control and DOX-treated mice did not differ, as we previously reported [16]. However, administration of PAESe, with or without DOX, significantly increased fEPSP amplitudes as stimulus intensities increased, with both PAESe groups exhibiting higher fEPSP amplitudes compared to DOX-treated animals at intensities of 0.06 and higher. This suggests that increasing basal synaptic transmission may be one mechanism by which PAESe rescues DOX-mediated deficits in synaptic plasticity and memory.

To determine whether the reduction in LTP was associated with presynaptic changes, we next measured the paired-pulse facilitation (PPF) ratio (Fig 2D). The PPF ratio (slope 2/slope 1) measured at different interpulse intervals was similar for all groups, indicating no significant difference in presynaptic release potential (*F*s < 2.9, *p*s > .12). Furthermore, the average fiber volley (FV) amplitude for each stimulus intensity was similar across all groups (Fig 2E, *F*s < 3.1, *p*s > .1), suggesting that the DOX-mediated changes in synaptic plasticity were not due to changes in axon recruitment.

### 3.3. Effect of doxorubicin on hippocampal protein levels and phosphorylation

**Fig 3:**
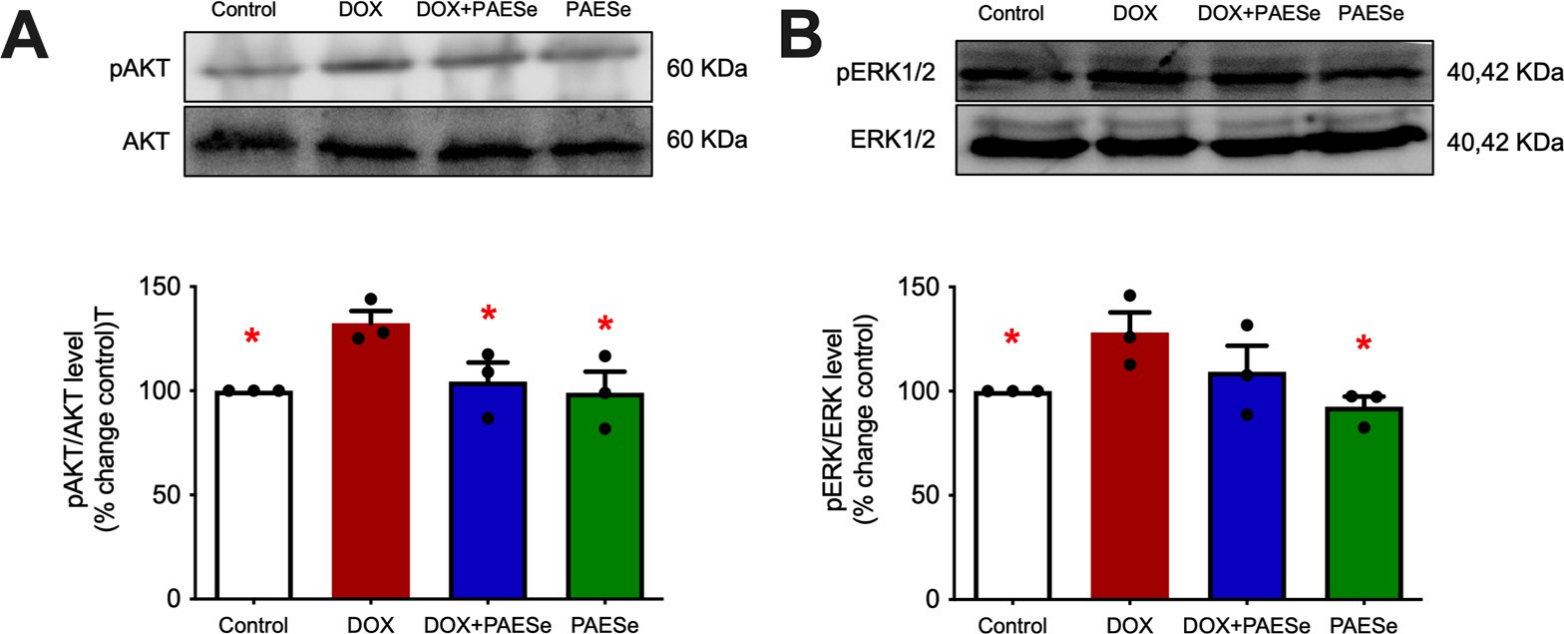
Doxorubicin affects phosphorylation of proteins required for synaptic plasticity. Representative immunoblot showing (**A**) pAKT/AKT and (**B**) pERK/ERK. Bars represent mean ± SEM; n = 3/group, * = *p* < .05 represents a significant difference when compared to the DOX group.

We have previously demonstrated [15,16] that mice treated with DOX exhibit increased phosphorylation of AKT and ERK1/2, two proteins vital for the maintenance of synaptic plasticity [33,34]. Thus, we sought to determine whether PAESe can reduce this DOX-induced increase in phosphorylation. We first examined total protein levels for AKT and ERK1/2 and observed no differences (Fig 3A and 3B). Consistent with our prior studies, DOX increased phosphorylation of AKT, and PAESe significantly reduced the DOX-induced increase in pAKT (Fig 3A; DOX effect, *F*(1,8) = 6.5, *p* < .05). DOX also increased phosphorylation of ERK1/2, though pERK1/2 was only reduced moderately by PAESe co-administration (Fig 3B; DOX effect, *F*(1,8) = 7.4, *p* < .05).

## 4. Discussion

The effects of DOX as a chemotherapeutic agent have been well investigated (see [5] for review). DOX binds to topoisomerase II, thus arresting the cell cycle and causing the death of tumor cells. In addition, DOX increases the production of reactive oxygen species, which causes cell death by damaging DNA and inducing lipid peroxidation. However, because of its mechanisms of action, DOX also causes adverse effects on healthy tissues and, as a consequence, DOX treatment is associated with several side effects, such as fatigue, inflammation, hypothyroidism, pain, discomfort, and cognitive impairment (the latter being known as “chemobrain”). In the present study, we used an immunocompromised mouse model of chemobrain [16] and drug dose to assess our hypothesis that the antioxidant PAESe, which has proven to provide cardioprotective benefits when co-administered with DOX [17], could protect against DOX-induced cognitive and synaptic impairments. Our findings suggest a significant disruption in hippocampal plasticity and memory in DOX-treated animals, which is ameliorated by PAESe co-treatment.

In this study, DOX was administered to male athymic NCr nude (nu/nu) mice at 5 mg/kg weekly for 4 weeks. It should be noted that greater doses of DOX have been associated with significant weight loss, lethargy, and increased mortality. Although this dosing regimen did not impair exploratory behavior, it caused impaired spatial memory as assessed by the Y-maze task. Importantly, the spatial memory impairment associated with DOX treatment was attenuated by the co-administration of PAESe. A similar observation resulted from the Novel Object Location test, in which DOX-treated animals explored less and showed lowered discrimination of a known object moved to a novel location, and PAESe treatment ameliorated these effects. It is unclear if the slight, but not significant, reduction observed in the novel objection location and percent exploration test observed with the administration of PAESe alone is associated with variation or pharmacologically mediated. Taken together, these data suggest that DOX treatment can disrupt memory processes that rely on intact hippocampal function and that PAESe rescues this dysfunction.

To investigate the effects of DOX and PAESe on hippocampal synaptic plasticity, we induced LTP in hippocampal slices acquired from each treatment group. We observed that LTP was impaired in slices obtained from DOX-treated animals compared to control mice, and that PAESe ameliorated DOX-induced LTP impairments. The DOX-induced impairment in LTP could be due to alteration of either the presynaptic release or the postsynaptic responses. Therefore, our study assessed axonal depolarization by measuring the FV amplitude at various stimulus intensities. This assessment indicated that the FV amplitude was not significantly different among groups, suggesting that the conversion of the presynaptic stimulus into axonal depolarization was not significantly affected by DOX, DOX+PAESe, or PAESe treatment compared to control animals. Moreover, an assessment of PPF, which measures the presynaptic response, revealed no significant differences across groups. For assessment of basal synaptic transmission, the average fEPSP slopes of the DOX+PAESe-treated and PAESe-treated animals were increased significantly relative to the DOX group at 0.06, 0.08, and 0.10 mA, suggesting an increase in glutamatergic signaling associated with PAESe treatment. Overall, our data suggest that the deficits in LTP due to DOX treatment are likely due to postsynaptic mechanisms, including an increase in basal synaptic transmission, and that PAESe ameliorates the LTP deficits induced by DOX, possibly *via* alterations in glutamatergic signaling.

In this study, we also examined several proteins associated with learning and memory, namely, Akt and ERK1/2. Akt and ERK1/2 signaling is required for normal neuronal development, function, and synaptic plasticity [35–39], and their phosphorylation is an important signal for synaptic plasticity and memory encoding [38,39]. While increases in phosphorylation of these proteins are typically associated with improvements in synaptic plasticity and memory, prolonged exposure to DOX has been linked to an increase in Akt and ERK1/2 signaling in the brain and in other tissue [40–46]. This excessive activation can increase oxidative stress, cell death, and oncogenic senescence [47]. Thus, we sought to determine whether PAESe co-administration would alleviate this DOX-induced increase in Akt and ERK1/2 phosphorylation. Though pERK1/2 was only moderately reduced by PAESe co-administration, Akt phosphorylation was significantly decreased following PAESE co-administration. This suggests that PAESE may alleviate oxidative stress associated with elevated levels of Akt phosphorylation, which may provide one mechanism by which PAESe ameliorated DOX-induced synaptic deficits and memory impairment.

## 5. Conclusions

With advances in early detection and aggressive treatment of primary cancer and metastatic disease, there are growing concerns associated with the development of symptoms associated with chemobrain. This study utilized behavioral, electrophysiological, and biochemical assays to assess the therapeutic potential of PAESe against DOX-induced chemobrain at drug concentrations that have been shown previously to be cardioprotective and improve antitumor activity in combination with DOX. Importantly, we observed that the antioxidant PAESe may ameliorate DOX-induced synaptic deficits and memory impairment when co-administered with DOX, indicating the potential therapeutic role for PAESe in chemotherapy-induced memory loss. Based on these promising results and existing knowledge, further investigation of different dosages and dosing schedules is warranted. These data should provide additional mechanistic insights and facilitate the optimization of therapy strategies for prevention and treatment of chemobrain, and possibly other CNS disorders.

## Supporting information

S1 Data (XLSX)

S1 Raw images (PDF)
