# Supplementary material for: Phenyl-2-aminoethyl selenide ameliorates hippocampal long-term potentiation and cognitive deficits following doxorubicin treatment
Source: PLoS One. 2023 Nov 10;18(11):e0294280. doi: 10.1371/journal.pone.0294280 (PMC10637675; doi:10.1371/journal.pone.0294280)
Supplement: S1 Raw images — (PDF) [file pone.0294280.s002.pdf]

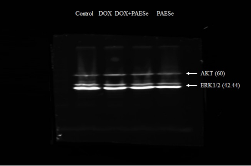
Control DOX DOX+PAESe PAESe
AKT (60)
ERK1/2 (42.44)

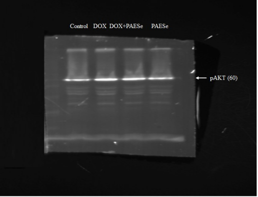
Control DOX DOX+PAESe PAESe
pAKT (60)

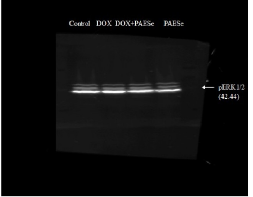
Control DOX DOX+PAESe PAESe
pERK1/2
(42.44)

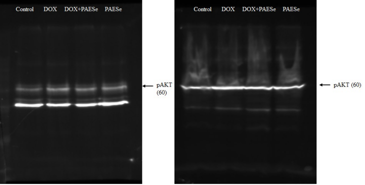
Control
DOX
DOX+PAESe
PAESe
pAKT
(60)
Control
DOX
DOX+PAESe
PAESe
pAKT (60)

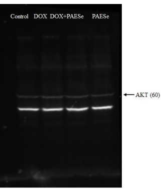
Control DOX DOX+PAESe PAESe
AKT (60)

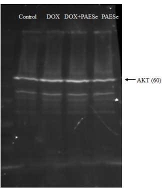
Control DOX DOX+PAESe PAESe
AKT (60)

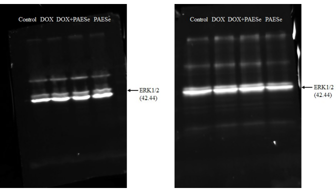
Control DOX DOX+PAESe PAESe
ERK1/2
(42.44)
Control DOX DOX+PAESe PAESe
ERK1/2
(42.44)

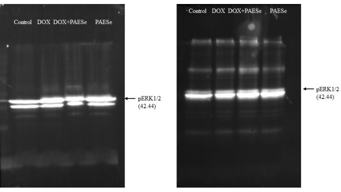

Control DOX DOX+PAESe PAESe
pERK1/2
(42.44)
Control DOX DOX+PAESe PAESe
pERK1/2
(42.44)

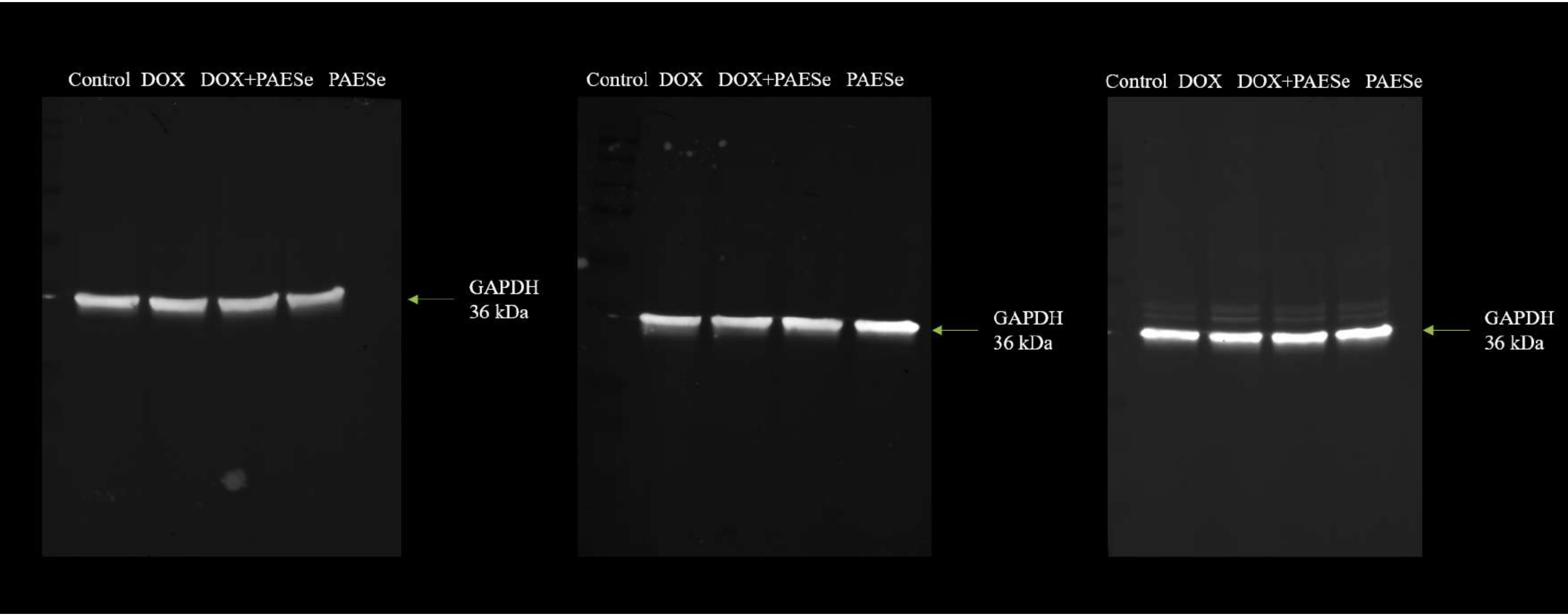
Control DOX DOX+PAESe PAESe
GAPDH
36 kDa
Control DOX DOX+PAESe PAESe
GAPDH
36 kDa
Control DOX DOX+PAESe PAESe
GAPDH
36 kDa
